# Problematic internet use among people with dentofacial deformity: a preliminary study

**DOI:** 10.3389/fpsyt.2025.1481739

**Published:** 2025-03-05

**Authors:** Marta Kożybska, Justyna Szpyt, Kacper Pajor, Iwona Radlińska, Anna Wojtkowska, Beata Karakiewicz

**Affiliations:** ^1^ Subdepartment of Medical Law, Department of Social Medicine, Pomeranian Medical University in Szczecin, Szczecin, Poland; ^2^ Student Research Group, Independent Subdepartment of Medical Law, Department of Social Medicine, Pomeranian Medical University in Szczecin, Szczecin, Poland; ^3^ Institute of Psychology, SWPS University, Wroclaw, Poland; ^4^ Subdepartment of Social Medicine and Public Health, Department of Social Medicine, Pomeranian Medical University in Szczecin, Szczecin, Poland

**Keywords:** problematic internet use, internet addiction, dentofacial deformity, class III malocclusion, malocclusion, young adults

## Abstract

**Objective:**

Class III malocclusion represents one type of anterior malocclusions, characterised by a longer face and a more prominent chin. Class III malocclusions are a type of malocclusion related to the relationship between the maxilla and mandible. They are often characterized by mandibular protrusion relative to the maxilla, and in some cases, features such as elongation of the lower facial third or a more prominent chin may also be present. Due to their appearance, patients experience a range of psychosocial and emotional difficulties, which have also been identified as risk factors for problematic internet use (PIU), including depression, negative body image, and lower self-esteem.

**Methods:**

A cross-sectional study was conducted and 170 fully completed questionnaires were obtained, 85 from people with Class III malocclusions aged between 18 and 42 years, and 85 from individuals without Class III malocclusions aged between 18 and 40 years.

**Results:**

Participants with Class III malocclusions obtained statistically significantly more PIU points than participants without Class III malocclusions (p < 0.001). Among people with Class III malocclusions 45.9% showed a high or very high risk of Internet addiction, while in the comparison group, it was only 9.4% (p < 0.001). Among participants with Class III malocclusions, there was a statistically significant, although weak, correlation between the PIU score and feeling uncomfortable when being the centre of attention (rho = 0.284; p < 0.01), and between the PIU score and concern with appearance (rho = 0.272; p < 0.05).

**Conclusion:**

Individuals with Class III malocclusion are at a much higher risk of problematic Internet use than people without the disorder. This problem especially concerns patients who feel discomfort when being the centre of attention and are more concerned about their appearance. Therefore, it seems that people suffering from disorders that cause changes in appearance should have access to extensive psychological support, including the prevention of problematic Internet use.

## 1 Introduction

The problem of Internet addiction has been a subject of interest to researchers around the world for years. To date, it has not been recognised as a separate disease. It is referred to as excessive Internet use, compulsive Internet use, or problematic Internet use (PIU). The latter refers to Internet use that results in difficulties in the domains of psychology, social interactions, educational performance and occupational functioning (1).

In 2016, Brand et al. published and then in 2019 updated The Interaction of Person-Affect-Cognition-Execution (I-PACE) model of specific internet-use disorders (2). The model describes the psychological and neurobiological processes underlying the addictive use of specific Internet applications. The model indicates predisposing variables, which include depression and social anxiety, feelings of isolation, loneliness, and low self-esteem (2, 3).

Other researchers have demonstrated that individuals with PIU exhibit a higher prevalence of interpersonal relationship difficulties (4–6), lower social skills (7), and social anxiety symptoms (8–11). Those who experience difficulty in forming interpersonal connections may be at an elevated risk of developing PIU (4–6). Moreover, individuals with PIU are more likely to experience feelings of loneliness, which, according to the most recent research, may be both a cause and a consequence of PIU (12). Furthermore, PIU is associated with a lower level of well-being (13).

Body image is also important in the development of PIU. The phenomenon of social media use and the rise in the number of people with body image disturbances have been increasing in recent years (14–17). Research indicates that negative body image perception is accompanied by an increase in social media addiction (18) and the risk of Internet addiction (19–23). At the same time, social media use was associated with women’s intentions to change their appearance (24). More exposure to edited photos on social media may increase the risk of negative self-perception (25). Individuals who more often compare their own appearance with the appearance of people they follow on social media are more prone to experiencing body dissatisfaction (26).

The problematic use of social media (e.g. Instagram) and associated mental health disorders, including loneliness, depression, anxiety and social anxiety, and body image dissatisfaction, are well described in the literature (27). Systematic review points to mental health disorders resulting from Problematic Instagram use (28). Children are particularly vulnerable to the negative mental health effects of excessive social media use (29). It can therefore be said that the PIU, particularly Problematic Use of Social Media, harms mental health.

As an aside, it should be added that there are also personality traits associated with PIU, particularly social media addiction, which include neuroticism (30–34), and also showed a negative relationship with conscientiousness (30–33) and agreeableness (30) Although there are cross-cultural differences in this area (35).

Many researchers identify young men (6, 36–49) as the population most at risk of developing PIU, as well as people using the Internet mainly for computer games (38, 42, 49), social networking sites, dating, and pornography (44). It should be noted that the vast majority of studies on the occurrence of PIU are conducted among teenagers and students. Meanwhile, it is worth identifying people also at risk of developing PIU in other groups in order to dedicate preventive programs to them. Taking into account the association of PIU with negative body image perception, problems with interpersonal relationships and loneliness, one of such groups may be people with facial deformation, e.g. with skeletal Class III malocclusions. Teenagers and young adults with facial deformities, such as Class III malocclusions, may face psychosocial challenges, such as negative self-image perception or interpersonal difficulties, which can potentially further increase the risk of PIU.Class III malocclusions are one of the types of anterior malocclusions that cause dentofacial deformity. The mandible is characterised by excessive forward growth, or the maxilla is not fully developed. The patient’s face is more oblong, and the chin is more protruding. These changes cause difficulties in chewing and biting food, and sometimes speech disorders (50–52), which may impair everyday functioning. Morphological prognathism is considered the most problematic maxillofacial deformity. The treatment methods include interceptive treatment (chin sling or facial mask), orthodontic camouflage, and surgical treatment, which brings the best results (53–55).

Individuals with Class III malocclusions experience many emotional difficulties (56), and are more prone to experience stress, symptoms of depression, and psychosocial difficulties (57, 58) resulting from the appearance of their faces (59). Mandibular prognathism is associated with lower quality of life and lower body image. Lee et al. showed that patients before orthognathic surgery were characterised by lower scores in terms of appearance perception and quality of life and higher scores in terms of appearance orientation and stigma of deformity compared to the control group (60). It has also been shown that patients undergoing orthognathic surgery have a worse body image than after surgery (61) and a lower quality of life (62).

Individuals with PIU and those with untreated Class III malocclusions experience symptoms of depression, psychosocial difficulties, worse body image, and lower self-esteem. Moreover, individuals with Class III malocclusions experience psychopathological correlates defined in Brand’s model (2, 3) as predisposing to the development of Internet addiction.

This suggests that the prevalence of PIU may be higher in individuals with Class III malocclusions compared to those without dental defects. Therefore, we formulated the following research hypotheses:

1. Individuals with Class III malocclusions are more likely to use the Internet in a problematic way than individuals without Class III malocclusions.
2. Individuals with Class III malocclusions who evaluate their appearance worse use the Internet in a more problematic way than individuals with Class III malocclusions who evaluate their appearance better.

This preliminary study aims to collect initial data justifying the further exploration of the relationship between PIU and dentofacial deformities, preliminarily verify the proposed hypotheses, perform an initial validation of the self-constructed questionnaire for Class III malocclusion patients, and assess the feasibility of a larger study.

## 2 Materials and methods

### 2.1 Study procedure and participants

The cross-sectional study was conducted as an anonymous survey in December 2018 and January 2019. Survey questionnaires for persons with Class III malocclusions were collected via the Internet by distribution to a Polish group of persons with Class III malocclusions on one of the social networks (Facebook). Individuals without Class III malocclusions were recruited for the study at three Polish universities with medical, technical, and economic education profiles (Medical University in Lublin, Silesian University of Technology in Gliwice, and the University of Economics in Katowice).

For data collection, we decided to use Google Forms, the most widely used survey tool in Poland at the time. The largest online group gathering individuals with Class III malocclusions in Poland is a group on one of the social media platforms, Facebook, named 'Progenia, retrognathism, and other malocclusions, before and after surgery photos! ):'. The group currently has 10.4 thousand members and includes individuals with various malocclusions and dentofacial disorders. The purpose of the group is to create a space for the exchange of patients' concerns and experiences. Membership in the group is granted to individuals who answer verification questions, such as whether they have one of the dentofacial disorders. Links to the survey for individuals with Class III malocclusions and no history of surgical treatment were posted in this group. Completing the survey took less than 10 minutes. The submission of incomplete surveys was blocked.

The study among students was conducted by trained interviewers using the paper-and-pencil method. Students were asked to complete the questionnaires after their classes in the lecture hall. They were informed about the purpose of the study and their right to withdraw at any stage. The surveys were completed anonymously, without any compensation, and participation was entirely voluntary. Those who did not wish to take part in the study were free to leave the room. Completing the survey took about 10 minutes.

We divided the inclusion criteria into three groups: (1) criteria common to all participants, (2) criteria specific to individuals with Class III malocclusions, and (3) criteria specific to individuals without Class III malocclusions.

1. The inclusion criteria common to individuals with and without Class III malocclusions were:
  - Age over 18 years. Only adults were included in the study. In Poland, where the study was conducted, the age of majority is 18.
  - Proficiency in the Polish language. Since the study was conducted in Poland, the questionnaire was prepared in Polish. Completing the questionnaire therefore required proficiency in Polish, which participants were informed about in the survey introduction.
  - Willingness to participate in the study. Participation was voluntary and anonymous. Participants were informed before completing the questionnaire that they could withdraw from the study at any time.
2. The criteria for individuals with Class III malocclusions were:
  - Existing Class III malocclusions. This information was self-reported by the study participants.
  - No history of surgical treatment for Class III malocclusions. This information was self-reported by the study participants.
  - Providing reliable and complete responses. Completing the questionnaire required answering all questions, as the system did not allow submission of incomplete forms. Additionally, responses were reviewed to check whether any participant had provided identical answers to all questions. No such cases were detected.
3. The criteria for individuals without Class III malocclusions were:
  - Absence of malocclusions. This information was self-reported by the study participants.
  - Aged between 18 and 42 years. This age range was selected to reflect the age distribution observed in the group with Class III malocclusions.
  - Providing reliable answers to the entire set of questions. The interviewer monitored the completeness of the questionnaires in real-time and only collected fully completed forms. No cases were recorded of questionnaires being completed in an unusually short time. Additionally, no instances of respondents providing identical answers to all questions in the questionnaire were detected.

The sample size was calculated at 71 using the EPI Infoprogram^TM^7. The prevalence of Class III malocclusions was estimated at 4.88% based on available literature data (53); the population of Poland is 37.73 million (63), confidence level was 95.0%, margin of error 5%.

The study design was submitted to the Bioethics Committee of the Medical University of Pomerania in Szczecin, which decided that the study did not have the characteristics of a medical experiment or clinical trial and therefore did not require the written consent of the participants (Decision No: KB-0012/188/05/17). Study participants were informed about the purpose of the survey, the possibility of withdrawing from the survey at any stage, and the aggregate publication of the results obtained. All the study participants were adults.

The study involved 170 participants, including 85 individuals with existing Class III malocclusions (not surgically treated), and 85 individuals without Class III malocclusions. In the group of participants with Class III malocclusions, the mean age was 24.55 years (SD = 5.36), with an age range from 18 to 42 years. In the group of participants without Class III malocclusions, the mean age was 23.85 years (SD = 4.65), with an age range from 18 to 40 years. Each group included 72 women and 13 men. The groups were not different in terms of age (Z = -1.098; p = 0.272) and had an identical gender distribution (**Table 1**). The control group was selected to ensure no statistical differences in age and to provide an identical gender distribution. To avoid differences in PIU within the group without Class III malocclusions that could result from the field of study [some research indicates higher PIU values among students of technical disciplines (42, 64)], the control group included 23 students of medical/health sciences, 29 students of humanities/social sciences, and 33 students of exact/technical sciences.

**Table 1:** Sociodemographic characteristics of study participants.

Variable	Persons with Class III malocclusions n=85	Persons without Class III malocclusions n=85
Age; M (SD)	24.55 (5.36)	23.85 (4.65)
Women	72	72
Man	13	13

*M*, mean; *SD*, standard deviation.

### 2.2 Measures

The study was conducted using the following tools:

#### 2.2.1 The problematic internet use test TPUI22

The questionnaire was used to measure problematic Internet use in both groups. It is a Polish adaptation (65) of the Internet Addiction Test developed by Dr Kimberly Young (66). The questionnaire consists of 22 questions that participants answer on a scale from 0 = ‘never’ to 5 = ‘always’. The maximum possible score is 110. The higher the score obtained, the greater the severity of problematic Internet use. The score obtained can be assigned to one of the following categories: very low risk of Internet addiction (0–1 points for individuals of ≤24 years and 0 points for those of >24 years), low risk of Internet addiction (2–10 points for individuals of ≤24 years and 1–6 points for those of >24 years), moderate risk of Internet addiction (11–49 points for individuals of ≤24 years and 7–41 points for those of >24 years), high risk of Internet addiction (50–79 points for individuals of ≤24 years and 42–75 points for those of >24 years), and very high risk of Internet addiction (80–110 points for individuals of ≤24 years and 76–110 points for those of >24 years). The tool has good reliability (Cronbach’s alpha = 0.935) (65).

#### 2.2.2 A purpose-written questionnaire covering feelings, behaviours, and a self-evaluation of the respondent’s appearance

This questionnaire was completed by individuals with Class III malocclusions. It is a self-administered questionnaire consisting of the following sets of questions:

- Feelings related to one’s appearance (how often do you feel frustrated, less confident, embarrassed, worried about your appearance, embarrassed about your appearance when meeting new people, feel uncomfortable about your appearance being the centre of attention). The respondents rated the frequency of each feeling on a scale of 1–5, with 1 – never; 2 – rarely; 3 – sometimes; 4 – often; 5 – very often;
- Behaviours related to one’s appearance (how often do you avoid smiling or laughing in company, hide your smile in photos, avoid looking at yourself in the mirror, compare your appearance to others, avoid meetings because you are unhappy with your appearance). Respondents rated the frequency of each behaviour on a scale of 1–5, with 1 – never; 2 – rarely; 3 – sometimes; 4 – often; 5 – very often;
- Appearance-rating questions (overall appearance rating, facial profile rating, frontal appearance rating, teeth appearance rating, nose appearance rating). The respondents rated these on a scale of 1–5, with: 1 – very bad; 2 – bad; 3 – neutral; 4 – good; 5 – very good.

The questionnaire was developed based on the available literature [among others (51, 56, 67, 68)], and other questionnaires with similar purposes. We were inspired by questions from the following instruments: the Oral Health Impact Profile-49 (OHIP-49) by Slade & Spencer (69) and the Orthognathic Quality of Life Questionnaire (OQLQ) by Cunningham et al. (70). The OHIP-49 is designed to measure the perception of the social impact of oral disorders on life, while the OQLQ assesses the quality of life in patients with dentofacial disorders. The OHIP-49 inspired us to include questions about embarrassment, irritation, and avoiding social interactions, whereas the OQLQ inspired the formulation of questions about hiding one's smile when taking photos, feeling embarrassed about appearance when meeting new people, and self-confidence. Moreover, one of the authors of our self-designed questionnaire was an individual with a history of Class III malocclusion, so the selection and construction of questions was based on suggestions from personal experience of this individual's condition. The authors also examined concerns about appearance expressed since 2017 by members in one of the groups on social media for individuals with Class III malocclusion.

The questionnaire was initially validated on a group of 161 patients with progenia (aged 18 to 47 years, M=25.81; SD=5.76; 87.6% women). The collected data were suitable for the factor analysis [KMO(df=120)=0,918; p<0,001]. Exploratory factor analysis revealed that the total score of 16 test items explains 63% of the total variance of the results with very good measurement reliability (Cronbach's alpha = 0.937), and suggested possible the two-factor structure:

- feelings and behaviours (11 items, explaining 49.2% of the total variance of the results; Cronbach's alpha = 0.941);
- appearance (5 items, explaining 13.9% of the total variance of the results, Cronbach's alpha = 0.806).

Factors were independent in structure and negatively correlated (rho = -0.601; p<0.001).

#### 2.2.3 A questionnaire of socio-demographic data.

In addition, a self-administered questionnaire with basic socio-demographic data was completed by both groups. The participants indicated the gender with which they identified most.

### 2.3 Statistical analysis

Statistical analysis was performed in the IBM SPSS Statistics v. 29 package and in the licensed Statistica 13.1 package. The Pearson’s chi-square test with additional Fisher-Freeman-Halton test (X2) was used to compare frequencies of categories of qualitative variables, the Mann–Whitney U test (Z) to compare mean scores of quantitative variables in two independent groups, and the pairwise correlation method with Spearman’s rho coefficient for variables with non-normal distributions. Only nonparametric methods were used because the data distribution did not meet the assumption of normality. A value of p < 0.05 was used as an indicator of statistical significance.

## 3 Results

The compared groups of participants with and without Class III malocclusions differed in the PIU score and PIU categorical results. **Table 2** presents the minimum, maximum, and average PIU score results. The Mann–Whitney U test showed a statistically significant difference between the PIU score among participants with and without Class III malocclusions (Z = -8,468; p < 0.001). The groups differed especially in the minimum scores – the lowest score achieved in the PIU test among people with Class III malocclusions was 22 points, while in the group without Class III malocclusions, there were participants who received 0 points. The average score among people with Class III malocclusions was 27 points higher.

**Table 2:** PIU in participants with and without Class III malocclusions.

All participants, N=170		Participants with Class III malocclusions, n=85		Participants without Class III malocclusions, n=85		Mann–Whitney U test	
Min–Max	M (SD)	Min–Max	M (SD)	Min–Max	M (SD)	Z	p
0 – 108	35.41 (23.88)	22 – 108	48.91 (19.47)	0 – 97	21.91 (19.98)	-8.468	<0.001

*Min-Max,* minimum – maximum*; M*, mean*; SD*, standard deviation.

**Table 3** shows the PIU categories. In the group of participants with Class III malocclusions, there were no people with a very low or low risk of Internet addiction. Meanwhile, as many as 25.9% of people in the group without Class III malocclusions belonged to these two categories. Although an average risk of Internet addiction prevailed in both groups, among people with Class III malocclusions, as many as 45.9% showed a high or very high risk of Internet addiction, while in the comparison group it was 9.4%. These differences were significant [X^2^(4) = 48,729; p < 0.001].

**Table 3:** Prevalence of PIU categories in participants with and without Class III malocclusions.

PIU category	All participants, N=170		Participants with Class III malocclusions, n=85		Participants without Class III malocclusions, n=85		Chi-squared test		
	N	%	N	%	N	%	X^2^	df	p
very low risk of Internet addiction	3	1.8	0	0.0	3	3.5	43.852	4	<0.001
low risk of Internet addiction	19	11.2	0	0.0	19	22.4			
moderate risk of Internet addiction	101	59.4	46	54.1	55	64.7			
high risk of Internet addiction	36	21.2	31	36.5	5	5.9			
very high risk of Internet addiction	11	6.5	8	9.4	3	3.5			

Gender did not differentiate PIU, neither in the entire group (Z = -0.078; p = 0.938), nor among the participants with Class III malocclusions [X2(2) = 0.341; p = 0.843], or the participants without Class III malocclusions [X2(4) = 3.934; p = 0.415]. PIU was also not correlated with age, neither in the entire group (rho = -0.001; p = 0.990), nor among the group with (rho = -0.158; p = 0.149), or without Class III malocclusions (rho = -0.060; p = 0.587). Gender and age did not influence the relationship between PIU and appearance assessment among patients.

**Table 4** presents the data on feelings, behaviours and evaluations related to one’s appearance among the participants with Class III malocclusions. There was a statistically significant, although weak, correlation between the PIU score and feeling uncomfortable when being the centre of attention (rho = 0.284; p < 0.01) and between the PIU score and concern with appearance (rho = 0.272; p < 0.05). Accordingly to possible two-factor structure of the questionnaire, PIU was positively correlated only with the first factor of feelings and behaviours. With the increase in the scores on this scale, the intensity of PIU also increased (rho= 0.257; p=0.017).

**Table 4:** Opinions of participants with Class III malocclusions about their appearance and Spearman’s correlation with PIU scores.

Variable		Participants with Class III malocclusions				
		M	SD	Mdn	Q3-Q1	r_s_
Feelings related to appearance*	frustration	3.62	1.16	4	2	0.126
	less self-confidence	3.73	1.26	4	2	0.140
	embarrassment	3.38	1.27	4	1	0.148
	concern with appearance	3.76	1.32	4	2	0.272^b^
	embarrassment with appearance when meeting new people	3.25	1.33	4	2	0.207
	discomfort in the centre of attention	3.51	1.31	4	2	0.284^a^
Behaviours related to one’s own appearance*	avoiding a smile or laughing in company	3.32	1.50	4	3	0.193
	hiding a smile in photos	3.76	1.43	4	2	0.105
	avoiding looking at yourself in the mirror	2.31	1.25	2	2	0.163
	comparing your appearance to others	3.86	1.38	4	2	0.208
	avoiding meetings due to dissatisfaction with your appearance	2.32	1.40	2	2	0.187
Appearance evaluation**	general appearance evaluation	2.86	0.93	3	1	-0.009
	facial profile evaluation	1.93	0.91	2	1	-0.171
	facial evaluation from the front	2.98	1.01	3	2	0.036
	teeth appearance evaluation	2.47	1.05	2	1	0.049
	nose appearance evaluation	3.12	1.29	3	2	-0.204

*rating on a scale of 1–5, a higher score means a more frequent occurrence of the feeling/behaviour,

**rating on a scale of 1–5, a higher score means a better assessment of the appearance,

^a^Correlation is significant at 0.01 (bilateral), ^b^Correlation is significant at 0.05 (bilateral),

*M*, mean; *SD*, standard deviation; *Mdn*, median; *Q3-Q*, interquartile range.

## 4 Discussion

To the best of our knowledge, our research is the first to address the problem of Internet addiction among people with appearance changes by disorder. Our results indicate that problematic Internet use was significantly more prevalent in individuals with Class III malocclusions than those without the condition. This suggests that persons with appearance dysfunctions are a PIU risk group and should receive appropriate PIU prevention support.

There are few studies indicating a link between PIU and physical diseases. In a 2018 study by Do and Lee (71), the high risk group of PIU had an increased experience of oral disease symptoms. In a 2021 study by Condori-Meza et al. (72), PIU was associated with Symptomatic Dry Eye Disease in medical students from Peru, but only in the male students. In a study of adolescents with a cancer diagnosis, PIU reached the highest rate among other variables (substance dependence, and other behavioural addictions) (73). These studies point to the fact that physical illnesses of varying severity and risk are linked to mental health. Of course, the issue is complex and concerns not only the risk of PIU but also other addictions (e.g. medication), which are closely linked to mental functioning (anxiety, depressive states). There are numerous studies on the role of depression in worsening health outcomes in chronic diseases (74). Also, patients with physical disorders are at a higher risk of depression. Monitoring such patients for depression is therefore recommended (75). Researchers report psychological intervention can prevent mood disorders and also treat them effectively (76). Also, it seems that mental health apps can be promising for reducing symptoms of mood disorders, especially depression (77). However, given the risks associated with PIUs (particularly those associated with phone use), caution should be exercised in the use of mobile applications.

The results of our study suggest that individuals with Class III malocclusion should have access to health education regarding PIU and the healthy use of technology.

There are numerous studies on the links between PIU and mental health, but they are inconclusive. In a 2018 meta-analysis (78), social network use was positively correlated with both positive and negative mental health indicators, with no significant difference between the two mean correlations. Considering cultural differences, stronger correlations between social network use and positive mental health indicators were found in collectivist cultures than in individualist cultures. In a study of Chinese adolescents by Chen et al., problematic internet use partially mediated the associations between adverse childhood experiences and health-related quality of life in adolescents (79). However, according to a systematic review, Internet activity can also enhance well-being and sustain healthy lifestyle behaviours (80). It is a fact that the Internet is nowadays used not only as a tool for entertainment, work, and education but also as a health care tool for users of broader health applications known as eHealth. At the same time, the need to introduce education on the prevention of PIUs and applications regulating the time of use of even health apps is emphasised (80). Numerous studies indicate the association of PIU with negative psychological phenomena such as depression, negative affect, stress (81), lower social skills (7), social anxiety symptoms (8–11), and loneliness (12).

The results of our study did not reveal significant statistical differences between gender and PIU in groups with and without Class III malocclusion. In global literature, numerous studies indicate that men are more likely to exhibit PIU than women (6, 38–43, 45–49, 82). However, some researchers suggest that gender does not significantly influence PIU (42, 83, 84). The relationship between gender and PIU may be linked to cultural differences among studied populations. Some researchers argue that social, economic, and cultural factors can cause differences in PIU, causing results from one population may not apply to another (35, 44, 85).

The differences in PIU levels between women and men may also result from the primary purpose of using the Internet. A systematic review suggests that an activity particularly prone to Internet addiction is online gaming (81). This aligns with the theory of specific addiction to the Internet, which refers to addiction to a specific online activity (3). At the same time, some studies indicate that men more often use the Internet for entertainment purposes like gaming, while women use it to communicate with others (43, 86–88). Therefore, men may potentially exhibit PIU more frequently due to the more addictive aim of their Internet use, but this needs to be confirmed in cross-population studies. Some studies suggest that the differences in PIU outcomes between women and men are related to specific aspects of the phenomenon (89, 90). Thus, the role of gender in generalized Internet addiction may be differ from its role in, for example, social media use.

Our study showed a statistically significant, albeit weak, correlation of PIU with feeling uncomfortable in the centre of attention and with concern for one’s appearance. No such correlation was noted in the other dimensions of attitudes related to one’s appearance (behaviour and assessment of appearance). In our study, among the Class III malocclusions subjects, as many as 45.9% showed a high or very high severity of PIU. This suggests a negative impact of this health problem, in particular body image, on the development of PIU. Studies by many authors point to the strong and negative impact of the Internet on body image. First and foremost, the disproportionate dependence of popularity on physical appearance in the online world is indicated (22, 91). Meanwhile, the images presented on social media platforms often deviate from the real ones – users present embellished versions of themselves through manipulation with filters and editing tools, leading to exaggerated standards of beauty and body (15). Unfortunately, such created images affect how young women (92). According to a 2019 systematic review, participants who spent more time on social media had a greater tendency to feel dissatisfied with their bodies, compare themselves to others, and internalise unrealistic beauty standards. Recent research also supports a link between social media use and negative perceptions of one’s body (14, 15). This problem may be particularly exacerbated in those with dentofacial deformity and requires further research.

Class III malocclusions are a physical health disorder, but due to their specificity of changes in appearance, as discussed further in the introduction, they are closely linked to the mental health sphere (56–59). Another significant factor that may influence Internet use among individuals with Class III malocclusions is loneliness, identified as one of the risk factors for PIU (2). This relationship also requires confirmation in further studies.

### 4.1 Limitations

Our preliminary research provides the first results concerning problematic Internet use among people with dentofacial deformity. The study is an introduction to the search for predictors of problematic Internet use among among these individuals. Despite the contribution of the research to the development of knowledge about Internet addiction, the research is not free from limitations. The first limitation of the study is its self-reported nature. The presence of Class III malocclusion was not confirmed by a specialist, but was only declared by the study participants. Future studies should consider the degree and subtype of Class III malocclusion, as well as the impact of the disorder on the daily functioning of affected individuals. Moreover, when selecting the study and control groups, additional sociodemographic variables should be considered, such as socioeconomic status, education level, marital status, and occupation. Interpretation of the current results should be approached with caution due to potential confounding factors stemming from differences in the recruitment process—the group with Class III malocclusions was recruited online, while the group without Class III malocclusions was selected from an academic environment. Future studies should ensure better control over confounding variables such as socioeconomic status, occupation, and education.

Because of the preliminary nature of the study, only adults were included. Limiting the study group to adults allowed the collection of initial results in a way that did not violate the privacy of minors. The results obtained provide initial evidence of the need for more in-depth research in this area, which could include minors. Furthermore, restricting the study to adults ensured that participants had sufficient cognitive and emotional maturity to give informed consent and participate in the study without undue difficulty, in accordance with ethical research principles. However, considering the characteristics of Class III malocclusion and the specific vulnerability of adolescents to PIU, future studies should also include adolescents.

Another limitation of the study is the collection of data from individuals with Class III malocclusion online. In future research, participants should be recruited through in-person contact, including verification of the diagnosis by a specialist. Direct data collection by an interviewer would ensure better control over the quality of the gathered data.

In the future, the study should also be expanded to include variables related to body image, loneliness, and depression symptoms in order to indicate the PIU predictors in this patient group.

Considering the above limitations, this study should be regarded as a preliminary study, providing a basis and justification for conducting further, in-depth research on PIU in individuals with dentofacial deformity and for the initial validation of the self-constructed questionnaire for Class III malocclusion patients. Further studies should include a larger number of sociodemographic variables, involve adolescents, and ensure diagnosis verification by a specialist, which suggests that these studies should be conducted in person rather than online.

## 5 Conclusion

The findings broadened the understanding of the potential groups at risk of developing PIU. Individuals with Class III malocclusion are at a much higher risk of problematic Internet use than people without this dentofacial deformity. This problem especially concerns patients who feel discomfort when being the centre of attention and are more concerned about their appearance. Therefore, it seems that people suffering from disordersthat cause changes in appearance should have access to the prevention of problematic Internet use. These studies should be developed to identify predictors of PIU among individuals with dentofacial deformities.

## Data Availability

The raw data supporting the conclusions of this article will be made available by the authors, without undue reservation.
